# High-resolution MRI-based Radiomics and Machine Learning for Identification of Middle Cerebral Artery Vulnerable Plaques

**DOI:** 10.2174/0115734056429820260505203621

**Published:** 2026-05-14

**Authors:** Danfeng Zhang, Bin Jiang, Tong Fu, Hui Xu

**Affiliations:** 1 Department of Radiology, Nanjing First Hospital, Nanjing Medical University, Nanjing, Jiangsu, 210006, China; 2 Department of Urology, the Fourth Affiliated Hospital of Nanjing Medical University, Nanjing 210031, Jiangsu, China

**Keywords:** Radiomics, Machine learning, Middle cerebral artery, Plaques, High-resolution magnetic resonance imaging, Traditional features, ICAD

## Abstract

**Introduction:**

To construct a vulnerable plaque model utilizing high-resolution magnetic resonance imaging (HR-MRI) based radiomics features and traditional features.

**Materials and Methods:**

110 patients with unilateral middle cerebral artery (MCA) stenosis and available HR-MRI data were enrolled. 50 plaques exhibiting acute stroke signals within the stenotic MCA region were categorized into the vulnerable plaque group, whereas the remaining 60 plaques were assigned to the stable plaque group. A traditional model was constructed by calculating the radiological plaque features. Radiomics features from HR-MRI were employed to establish a vulnerable plaque model with a support vector machine (SVM). A combined predictive model was then built. The efficacy of these models was assessed using receiver operating characteristic curves.

**Results:**

The traditional model demonstrated an AUC of 0.852 in the training set and 0.839 in the test set. While the radiomics model and the combined model showed an improved AUC: 0.919 vs. 0.902 for the training sets and 0.955 vs. 0.949 in the test sets. Both the radiomics model and the combined model outperformed the traditional model in the two sets (*P*<0.05); the performance of the combined model was not significantly different from that of the radiomics model in both sets (*P*>0.05).

**Discussion:**

A combined model based on radiomics and traditional features improves the ability to differentiate the vulnerable MCA plaques from stable ones.

**Conclusions:**

The radiomics model derived from HR-MRI exhibits high accuracy in identifying the vulnerable plaques that are likely to cause acute stroke, and this model was significantly superior to the traditional model.

## 1 INTRODUCTION

Intracranial atherosclerotic disease (ICAD) is the leading cause of strokes worldwide, with a noteworthy prevalence among the Asian population [1, 2]. Although the exact disease mechanism is not fully understood, ICAD commonly affects different segments of blood vessels. In the Chinese population, ICAD is responsible for 30% to 50% of ischemic strokes, and the middle cerebral artery (MCA) is the most frequently affec-ted vessel in intracranial atherosclerosis by ICAD [3, 4]. Di-verse atherosclerotic plaques lead to different outcomes, with some causing symptoms and others remaining silent, making clinical management more challenging [5, 6]. Accurately assessing plaque characteristics and determining risk levels are essential for guiding effective clinical interventions.

High-resolution magnetic resonance imaging (HR-MRI) allows for thorough noninvasive evaluation of MCA atherosclerotic plaques, despite the challenges posed by small plaque size and the absence of histological validation for in vivo application [7,8]. Surface irregularity, T1-weighted imaging (T1WI) hyperintensity, and plaque enhancement have been identified as imaging biomarkers for ischemic events in symptomatic patients when evaluating plaque characteristics [9]. Meanwhile, many other scholars in the past have extensively described similar typical features and qualities of plaques, for instance, when compared to those without symptoms, individuals experiencing symptoms demonstrated larger plaque areas (PA), a higher occurrence of vessels with positive remodeling, and much more enhanced plaques [10,11]. However, images contain an adequate quantity of information beyond what can be easily visualized or quantified through basic manual assessments [12]. Advancements in computa-tional technologies have facilitated the transformation of radiomics into a plethora of quantitative features in an unbiased, consistent, and efficient manner [13].

Radiomics is a computer-aided technique used to derive quantitative characteristics from a large number of medical images, offering benefits like precision and freedom from observer bias [14,15]. There have been some meaningful applications of radiomics in coronary [16], carotid [17], and intracranial artery [12] recently. According to a predominant impact on the MCA in the Chinese population, we aimed to establish a vulnerable plaque model that utilized enhanced T1WI radiomics features in combination with machine learning, and then evaluated its performance by comparing it with the traditional model.

## 2 MATERIALS AND METHODS

### 2.1 Subjects and Clinical Data

The study was conducted in accordance with the Declaration of Helsinki (as revised in 2013). The research protocol was approved by the Ethics Committee of Nanjing Medical University (No. NMU20200320). All patients included in the study provided their informed consent, either verbally or in written form. A total of 110 consecutive individuals who were admitted for ischemic stroke or transient ischemic attack, and suspected of having MCA atherosclerotic diseases, were enrolled from the Department of Neurology,from January 2022 to June 2024. Patients were included in the study based on the following criteria: (1) presence of more than two risk factors for atherosclerosis (for example: hypertension, hyperlipidemia, diabetes, smoking, obesity, family history) ; (2) absence of contraindications for MR scans; (3) unilateral stenosis in the M1 segment of the middle cerebral artery, ranging from 50% to 99% as visualized on enhanced magnetic resonance angiography (MRA); (4) absence of significant stenosis (≥ 50%) in the ipsilateral internal carotid artery (ICA); (5) no presence of non-atherosclerotic vascular disorders like brain hemorrhage, vasculitis, moyamoya disease, dissection, or tumors; (6) lack of evidence suggesting the presence of atrial fibrillation or cardioembolism; (7) high-quality images appropriate for diagnosis and assessment; and (8) confirmation that the M1 segment of the MCA being studied is in a straight configuration on enhanced MRA. Individuals who do not comply with the above 8 inclusion criteria are excluded and will not be included in the research. In this study, the power analysis was performed, and the sample size was determined by the “PS: Power and Sample Size Calculation” software (by William D. Dupont and Walton D. Plummer, Jr), which could calculate statistical power or sample size for a clinical study.

Patient demographic and clinical information, such as gender, age, smoking status, alcohol intake, hypertension, and diabetes status, alongside blood glucose, HbA1c, total cholesterol, LDL cholesterol, HDL cholesterol, triglyceride, and homocysteine levels, were gathered from their medical records.

### 2.2 MRI Protocol

One week after admission, the patients underwent standard MRI scans including axial T1-weighted imaging (T1WI), T2-weighted imaging (T2WI), T2-fluid attenuated inversion recovery (FLAIR), diffusion-weighted imaging (DWI), apparent diffusion coefficient (ADC), and enhanced MRA. The stenotic MCA was imaged using a 3-Tesla MR scanner (Ingenia, Philips Medical Systems, Netherlands) equipped with an 8-channel head coil. Then, contrast-enhanced T1WI (CE-T1WI) perpendicular to the MCA was performed throughout the whole brain with specific scan parameters: TE of 19 ms, TR of 800 ms, slice thickness of 0.8 mm, no slice gap, 200 slices, number of excitation: 2, matrix size of 252 mm x 228 mm, and field of view (FOV) of 200 mm x 80 mm x 181 mm, acquisition time: 6 minutes. A 15 ml bolus of gadodiamide was administered intravenously at a speed of 2 ml/s, followed by the acquisition of T1WI+C five minutes after the injection of the contrast agent.

### 2.3 Traditional Features Measurement

Vessel wall parameters were measured using image-based methods on the Philips Intellispace Portal workstation. Manual measurements of lumen area (LA) and vessel area (VA) were conducted on enlarged (300%) short-axis CE-T1WI images at both the reference segment (selected from the contralateral MCA) and the most narrowed lumen (MNL) of the MCA. Wall area (WA) was calculated as VA - LA, plaque area (PA) as WA_MNL_ - WA_reference_, remodeling index (RI) as VA_MNL_/VA_reference_, normalized wall index (NWI) as WA/VA, and degree of stenosis as (1 - LA_MNL_/LA_reference_) x 100%. These parameters were determined following established research [7, 18]. Plaques were categorized based on DWI and ADC signals within the stenotic MCA region: hyperintense signal on DWI and hypointense signal on ADC indicated vulnerable plaques, whereas the rest of the plaques were grouped as stable. Positive remodeling (PR) was defined as RI ≥ 1.05, non-remodeling as 0.95 < RI < 1.05, and negative remodeling as RI ≤ 0.95. Two blinded radiologists conducted the measurements, reaching a consensus on the chosen slices. Any discrepancies were resolved through joint readings to achieve agreement.

### 2.4 Radiomics Feature Extraction

The plaque was manually delineated using the open-source software ITK-SNAP (v3.8.0; www.itksnap.org), jointly completed by two radiologists. We used an interpolator of “sitk B Spline” to resample the images to an isotropic voxel size. To mitigate data heterogeneity bias, the images were resized to a resolution of 0.3 mm x 0.3 mm x 0.3 mm, and their intensities were standardized on a scale ranging from 0 to 100 before extracting radiomics features. For discretization, we used a bin width of 5. The number of bins was 20. We pre-trained the model on the Onekey platform for transfer learning. Handcrafted features can be categorized into three main groups: (I) geometry, which characterizes the 3D shape of the plaque; (II) intensity, focusing on the statistical distribution of voxel intensities within the plaque; and (III) texture, capturing spatial intensity patterns using methods like gray-level co-occurrence matrix (glcm), gray-level run length matrix (glrlm), gray-level size zone matrix (glszm), and neighborhood gray-tone difference matrix (ngtdm) [16].

### 2.5 Model Construction

Traditional model: the features utilized in constructing the traditional model were chosen based on radiological parameters with a significance level of *P* < 0.05. A 5-fold cross-validation and a fixed test cohort were employed to ensure fair comparison.

Radiomics model: The data were divided randomly into training and test sets, with 80% allocated to training and 20% to testing. Normalization using the Z-distribution was conducted on all radiomics features to standardize the data. We performed Mann-Whitney U tests and feature selection, retaining only radiomic features with *P* < 0.05. For highly reproducible features, Spearman's rank correlation coefficient was calculated, and one feature from any pair with a coefficient >0.9 was retained. A greedy recursive deletion strategy was then implemented to filter features, removing redundancies iteratively. The Least Absolute Shrinkage and Selection Operator (LASSO) regression model was employed on the discovery dataset to construct a signature. 5-fold cross-validation determined the optimal λ value with minimal error, selecting features with nonzero coefficients for model fitting. The retained features were integrated into the radiomics model after LASSO feature selection. Python's scikit-learn package facilitated LASSO regression modeling. Following this, the final features were fed into machine learning models such as Support Vector Machine (SVM), utilizing 5-fold cross-validation to generate the ultimate radiomics signature.

Combined model: the combined model was constructed using both the selected radiological variables and radiomics features, along with the corresponding receiver operating characteristic (ROC) curves. Performing decision curve analysis (DCA) to evaluate the clinical utility of predictive models.

## 3 STATISTICAL ANALYSIS

Statistical analyses were performed using the SPSS 21.0 software package (Chicago, IL, USA) and R software (version 4.2.2, www.r-project.org), and Python (version 3.9.7, www.python.org). The Kolmogorov-Smirnov test was utilized to assess the normality of quantitative data distribution. Normally distributed continuous data is often represented as mean ± standard deviation, while non-normally distributed data is described using the median and interquartile range (IQR). Continuous variables were compared using the t-test, while categorical variables were analyzed with the Pearson chi-square test. Non-normal data was assessed by the Mann-Whitney U test. Significant indicators were then subjected to multivariate logistic regression. *P* < 0.05 was considered significant. Inter-observer reliability for continuous variables was evaluated using intraclass correlation coefficients (ICC) with a two-way random effect model. A robust agreement among observers was suggested by an ICC greater than 0.75. The Mann-Whitney U test was used for statistical analysis and feature selection among all radiomic features, retaining only those with *P*-values < 0.05. Discriminative performance was evaluated through ROC curve analysis, with DeLong tests utilized for ROC comparisons. ICC was computed to assess both intra- and inter-observer agreements. Statistical significance was set at a two-tailed *P*-value < 0.05.

## 4 RESULTS

### 4.1 Demographic and Clinical Characteristics

After excluding 52 patients due to various reasons (24 with moving artifacts, 18 with ipsilateral ICA stenosis≥50%, 6 with brain tumor, and 4 with brain hemorrhage), the study enrolled a total of 110 participants as depicted in Fig. (**1**). Among them, 60 individuals belonged to the stable group, while the vulnerable group comprised 50 patients. Analysis of sex, smoking habits, alcohol consumption, hypertension, diabetes, age, blood glucose levels, total cholesterol, triglycerides, low-density lipoprotein (LDL), high-density lipoprotein (HDL), HbA1c, and homocysteine levels showed no statistically significant differences between the two groups (Table **1**).

### 4.2 Radiological Parameters of MCA Atherosclerotic Plaques

The univariate analysis results indicated that LA_MNL_, RI, PA, and NWI_MNL_ were statistically different between the two groups in both the train set and test set (*p*<0.05, Table **2**). Multivariate logistic regression analysis indicated LA_MNL_ (*P* = 0.000; 95% CI: 0.012-0.174), RI (*P* = 0.018; 95% CI: 1.146–2.145), and PA (*P* = 0.000; 95% CI: 2.136–5.877) were independent predictors of acute stroke occurrence within the MCA territory, which were included in traditional model analysis. The value of AUC in the train set and the test set was 0.852 and 0.839, respectively.

### 4.3 Radiomics Model and Combined Model Construction of MCA Plaques

After LASSO feature screening, we input the final 108 radiomics features, which were extracted from the ROI on the CE-T1WI sequence, into the machine learning model SVM for risk model construction. Consequently, only 8 features (y=0.13original_glszm_SizeZoneNonUniformity+0.original_shape_Maximum2DDiameterColumn+0.06original_ngtdm_Complexity+0.05original_firstorder_Maximum+0.04original_glszm_LowGrayLevelZoneEmphasis+0.04original_glszm_LargeAreaLowGrayLevelEmphasis+0.01original_gldm_DependenceEntropy+0.01original_shape_SurfaceVolumeRatio) were retained in the end. Radiomic feature weights were shown in Fig. (**2**).

**Fig. (3):**
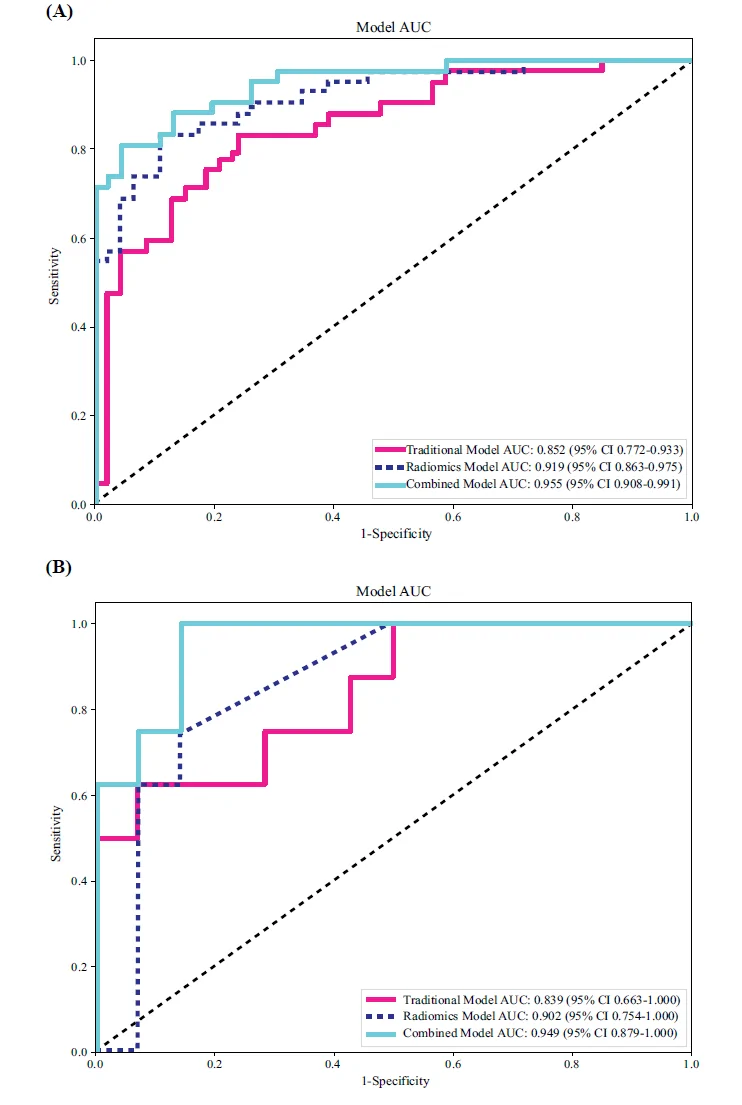
Receiver operating characteristic (ROC) curves of all the models. (**A**): AUCs of models in the training sets; (**B**): AUCs of models in the test sets.

Based on the radiomics, the model achieved AUC values of 0.919 and 0.902 in the training and test sets, respectively. When integrating traditional and radiomics features, the AUC values increased to 0.955 and 0.949 in both sets (Fig. **3**). Comparison of ROC curves revealed superior performance for both the radiomics and combined models compared to the traditional model in both the training (*P* = 0.000, *P* = 0.024) and test sets (*P* = 0.010, *P* = 0.009). Notably, no significant difference was observed between the radiomics and combined models in the two sets (*P* = 0.066, *P* = 0.545).

In this study, decision curve analysis (DCA) was also conducted to assess each model. DCA results for the traditional, radiomics, and combined models in both the training and test sets are depicted in Fig. (**4**). The combined model demonstrated superior clinical benefit compared to scenarios without any prediction model. Predictive ability of three models in two sets was shown in Table **3**. The Nomogram was displayed in Fig. (**5**).

### 4.4 The Inter-observer Agreement

The intraclass correlation coefficients (ICCs) for evaluating the traditional and radiomics features varied between 0.835 and 0.947.

## 5 DISCUSSION

In this retrospective study, we built a combined model based on 8 CE-T1WI radiomics features (2 shape features, 1 first-order feature, and 5 texture features) and traditional risk factors (LA_MNL_, RI, and PA) that improves the ability to differentiate the vulnerable MCA plaques from stable ones.

The research on intracranial arterial plaques has evolved from simple measurements in the beginning to a later emphasis on the importance of plaque composition for stability assessment. The pathology acquisition of intracranial arterial atherosclerotic plaques was difficult [6,10], so scholars presumed plaque composition by studying the signals of the plaques. Then, many studies have been conducted to investigate relevant plaque components or characteristics in relation to the risk of ipsilateral acute stroke events attributed to ICAD atherosclerosis [7,9,10,19]. The high signal within the plaque on T1-weighted imaging was considered to be intraplaque hemorrhage, which could easily lead to stroke [17,19]. Enhancement of plaques was believed to indicate the presence of a significant number of newly formed capillaries and infiltrating inflammatory cells inside, making them prone to rupture and causing symptoms [20]. Since the plaque stability is a critical factor in stroke prevention and treatment, extracting more valuable information from existing image data has become increasingly significant.

Radiomics has emerged as a technique for offering quantitative data that is noninvasive and independent of human interpretation, addressing the constraints of visual evaluation [21]. In order to overcome radiomics' limited robustness and reproducibility, improving radiomics standardization concerns, Mayerhoefer *et al.* emphasized that MRI scans with increased resolution exhibit improved classification accuracy [22]. Higher image resolution is more conducive to reliable radiomics information extraction. In this study, before extracting omics features, the raw images were resampled to 0.3mm*0.3mm*0.3mm, representing the thinnest layer of research known to date on plaques in the head and neck region [12,17,23]. Meddings Z *et al.* discovered that utilizing a multi-slice strategy for radiomics leads to enhanced robustness and predictive precision compared to employing a single-slice approach [24]. Therefore, we carefully outlined all the levels containing MCA plaques, incorporating more precise shape information of the plaques. In the results, we extracted meaningful features of two patch shapes, including surface volume ratio and maximum 2D diameter column, which demonstrated that the steeper the surface of the plaque, the more likely it is to cause a stroke, similar to a previous study [25]. In addition, since previous literature [20] has shown that the judgment of plaque characteristics by CE-T1WI is superior to plain scans, we have adopted the CE-T1WI sequence for radiomic feature extraction. 1 first-order feature, 5 texture features were also discovered in our research. First-order features refer to imaging features exhibited by individual voxels, while texture features refer to the presentation of textures shown by explicitly designated gray-level matrices, and each category of features indicates certain facets of the image, encompassing associations with neighboring voxels, voxel groupings, and linear dimensions. Of these 8 selected features, the original_glszm_sizeZoneNonUniformity feature had the highest weight coefficient. In the context of radiomics, the “original_glszm_sizeZoneNonUniformity” feature in imaging informatics refers to the measure of the variability of zone sizes for gray level size zone matrices in a medical image. It quantifies how uniform or varied the zone sizes are within the image, providing insights into the texture and spatial distribution of intensity levels within regions of interest; a higher value indicates greater heterogeneity in the sizes of zones with similar gray-level intensities, while a lower value suggests more consistent zone sizes in the image [26]. These texture features demonstrated that the CE-T1WI images of patients with stroke occurrence had more heterogeneity and larger contrast with adjacent voxels than those of patients without symptoms, which is indistinguishable by visual inspection. Additionally, our study constructed a combined model integrating radiological variables and radiomic features to enhance predictive performance. We used the SVM approach, which is suitable for high-dimensional and small sample data in radiomics. SVMs found an optimal boundary to distinguish disease states or predict outcomes and excel in handling nonlinear problems by mapping data to a high-dimensional space.

Considering the additional image information beyond what is discernible by the naked eye provided by radiomics, our research has found that radiomics models outperform traditional models. Compared with the traditional model, both the AUC value and the prediction accuracy of the radiomics model have been improved. Although this study only included 110 cases, all cases involved the M1 segment of the middle cerebral artery in the brain. This is the most commonly affected location by intracranial atherosclerosis in the Asian population, which easily leads to large-scale strokes,and the study also excluded factors related to different intracranial blood vessels to ensure the reliability of the results.

Our study faced some limitations. Firstly, although our reproducibility was great, we were unsuccessful in automatically segmenting ROIs. But we employed experienced radiologists to delineate the lesion areas in each image, ensuring the accuracy of the radiomics information. Secondly, our research was a retrospective study conducted at a single center, potentially introducing selection bias. External validation would be needed by using another cohort. Thus, to validate and expand upon our findings, it is essential to conduct prospective and multicenter longitudinal studies following standardized protocols. Thirdly, the relatively small sample size highlights the necessity for more data to enhance and validate the model in future research. Additionally, we used single-modality research, only outlining the CE-T1WI sequence. More sequences should be included in the future study. Fourthly, the state of the plaque changed over time. Our research defined the state of the plaque only based on the imaging manifestations at the time of the examination. Fifthly, manual delineation was associated with observer bias, and subsequent studies will attempt to adopt semi-automatic or fully automatic delineation methods to improve the reproducibility of the paper. Lastly, considering the higher incidence of intracranial atherosclerosis in Asian populations compared to European and American populations, further investigation is needed to assess potential ethnic-related differences.

## CONCLUSION

In this retrospective study, we built a combined model based on radiomics features and traditional risk factors, which improves the ability to differentiate the vulnerable MCA plaques from stable ones. Radiomics analysis of middle cerebral artery plaques on HR-MRI demonstrated strong reproducibility and enhanced capability in distinguishing between vulnerable and stable plaques, surpassing traditional features evaluation. Additional prospective studies will enhance clinical strategies for individuals at elevated risk in the future.

## Figures and Tables

**Fig. (1):**
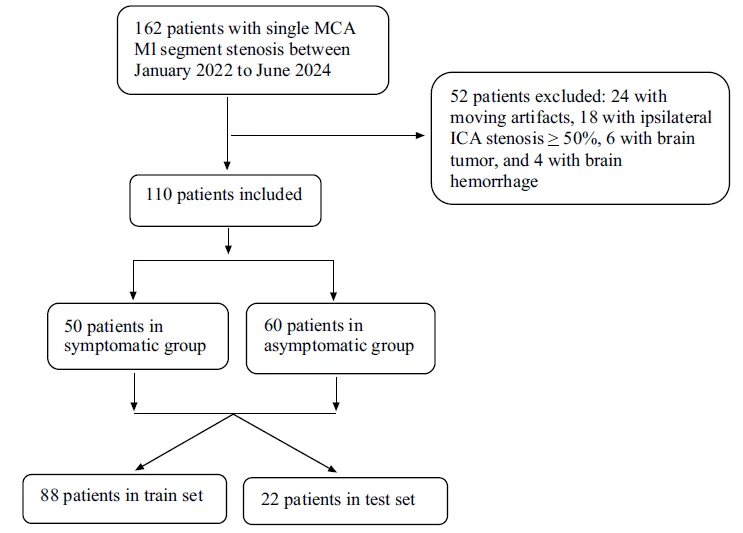
Study flow chart.

**Fig. (2):**
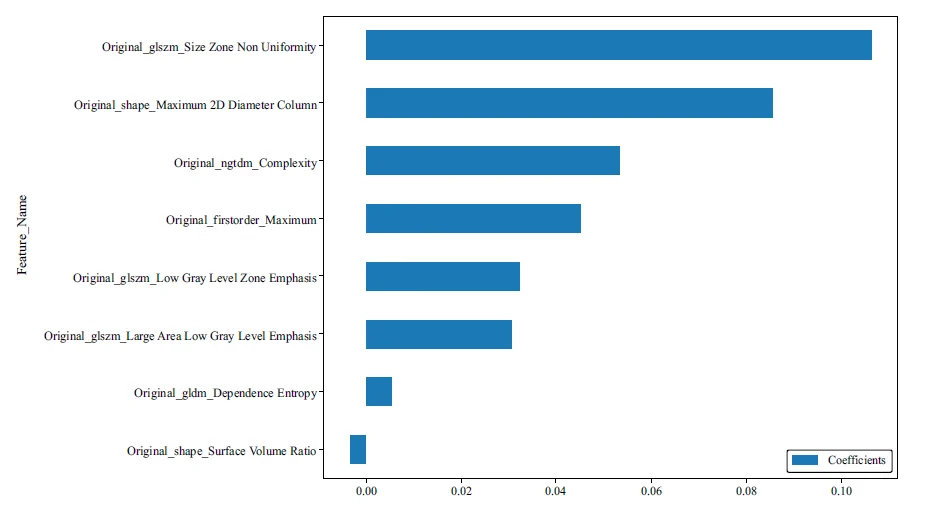
Radiomics features weights.

**Fig. (4):**
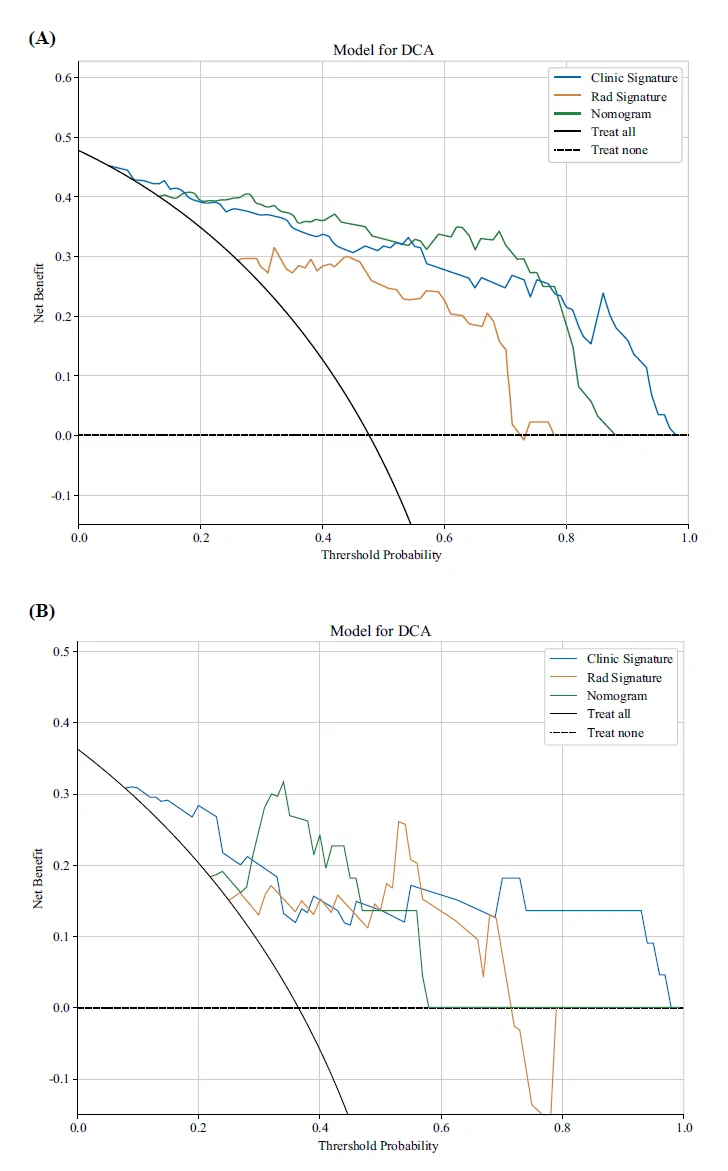
Decision curve analysis (DCA) for all models in two sets; the Y-axis shows the model benefit, the X-axis means the threshold probability. (**A**): DCA of models in the training sets; (**B**): DCA of models in the test sets.

**Fig. (5):**
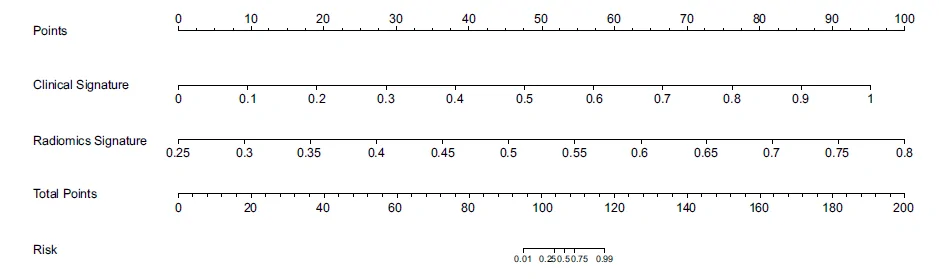
Nomogram.

**Table 1:** Demographic and clinical characteristics of the asymptomatic group and the symptomatic group.

-	Asymptomatic Group (n=60)	Symptomatic Group (n=50)	t-value	*P*-value
Sex (male)	29	27	-	0.554^a^
Smoker	32	30	-	0.426^a^
Alcoholism	22	24	-	0.230^a^
Hypertension	38	32	-	0.942^a^
Diabetes mellitus	26	28	-	0.186^a^
Age (years)	63±15	67±13	-0.79	0.483^b^
Blood glucose (mmol/L)	5.32±1.55	6.44±1.61	-1.18	0.227^b^
Total cholesterol (mmol/L)	4.71±1.28	4.88±1.25	-0.35	0.702^b^
Triglycerides (mmol/L)	2.39±1.32	2.81±1.13	0.67	0.566^b^
HDL (mmol/L)	0.99±0.45	1.29±0.53	-1.28	0.194^b^
LDL (mmol/L)	2.88±1.09	3.18±1.15	0.46	0.669^b^
HbA1c (%)	7.35±1.42	7.79±1.62	1.22	0.208^b^
Homocysteine (µmol/L)	12.74±3.87	13.91±3.59	1.40	0.114^b^

**Note:** The *P*-values are obtained by using the Pearson chi-square test. bThe P value is obtained by a t-test. HDL=High-density lipoprotein, LDL=Low-density lipoprotein. Data are presented as numbers (%) or means ± standard deviation.

**Table 2:** Radiological parameters of MCA atherosclerotic plaques in the train set and test set.

	Train Set (n=88)			Test Set (n=22)		
	Symptomatic Group(40)	Asymptomatic Group(48)	*P*-value	Symptomatic Group(10)	Asymptomatic Group(12)	*P*-value
VA_MNL_	14.08±3.30	13.86±2.94	0.739	16.20±3.49	12.93±2.34	0.290
LA_MNL_	2.39±0.79	3.68±0.89	0.000	2.46±0.70	3.36±0.98	0.029
VA_reference_	13.29±3.45	14.78±3.65	0.053	15.96±5.73	13.46±2.63	0.441
LA_reference_	6.93±2.07	8.26±2.35	0.106	7.55±1.24	7.72±1.87	0.823
RI	1.09±0.07	0.94±0.08	0.000	1.04±0.08	0.97±0.06	0.013
Sd(%)	66.21±8.88	67.04±8.26	0.490	67.25±8.70	64.14±8.90	0.437
PA	5.71±1.56	4.26±1.18	0.000	5.84±1.67	4.22±1.15	0.014
NWI_reference_	0.47±0.10	0.44±0.08	0.109	0.49±0.16	0.43±0.09	0.254
NWI_MNL_	0.85±0.04	0.79±0.03	0.000	0.86±0.05	0.78±0.05	0.000
WA_MNL_	11.71±2.92	11.17±2.52	0.353	12.71±3.62	10.18±1.87	0.094
WA_MNL_	6.36±2.34	6.52±2.14	0.751	6.41±2.42	5.74±1.60	0.402

**Note:** The *P*-value is obtained by a t-test. MNL=most narrowed lumen; reference= reference site; VA=vessel area; LA=lumen area; RI=remodeling index; Sd=stenotic degree; PA=plaque area; NWI=normalized wall index; WA=wall area.

**Table 3:** Predictive ability of all models.

Cohort	Signature	AUC	95%CI	Accuracy(%)	Sensitivity(%)	Specificity(%)	NPV(%)	PPV(%)
Train	Traditional	0.852	0.772-0.933	84.6	81.0	82.1	67.4	75.6
Train	Radiomics	0.919	0.863-0.975	90.1	88.3	86.6	83.7	87.2
Train	Combined	0.955	0.908-0.997	94.8	92.5	90.4	77.8	92.5
Test	Traditional	0.839	0.663-1.000	79.3	70.0	80.9	76.5	80.0
Test	Radiomics	0.902	0.754-1.000	88.4	82.5	85.7	92.3	77.8
Test	Combined	0.949	0.879-1.000	92.9	87.6	89.9	83.0	94.3

**Abbreviations:** AUC=area under the receiver operating characteristic curve; CI=confdence interval; NPV=negative predictive value; PPV=positive predictive value.

## Data Availability

All the data and supportive information are provided within the article.

## LIST OF ABBREVIATIONS

HR-MRI: High-Resolution Magnetic Resonance Imaging
MCA: Middle Cerebral Artery
SVM: Support Vector Machine
ICAD: Intracranial Atherosclerotic Disease
T1WI: T1-Weighted Imaging
PA: Plaque Areas
LA: Lumen Area
VA: Vessel Area
PR: Positive Remodeling
LASSO: Least Absolute Shrinkage And Selection Operator
ROC: Receiver Operating Characteristic
DCA: Decision Curve Analysis
IQR: Interquartile Range
ICC: Intraclass Correlation Coefficient
ICCs: Intraclass Correlation Coefficients

## STANDARDS OF REPORTING

STROBE guidelines were followed.
